# Integrative nanoformulation of paclitaxel, ruthenium (II), and curcumin for enhanced oral cancer cell suppression

**DOI:** 10.1016/j.jobcr.2025.10.024

**Published:** 2025-11-01

**Authors:** Rethinam Senthil, Deepak Angamuthu, P. Geetha Sravanthy, R. Pradeep Kumar

**Affiliations:** a Nano-Bioproduct Research Lab (NBRL), Department of Pharmacology, Saveetha Dental College and Hospitals, Saveetha Institute of Medical and Technical Sciences (SIMATS), Saveetha University, Chennai, 600 077, Tamilnadu, India; b Department of Radiation Oncology, SPECTRA Cancer Centre, Saveetha Medical College, Saveetha Institute of Medical and Technical Sciences (SIMATS), Saveetha University, Chennai, 600 077, Tamilnadu, India; c Department of Public Health Dentistry, Saveetha Dental College and Hospital, Saveetha Institute of Medical and Technical Sciences, Saveetha University, Chennai, India

**Keywords:** Complex drugs, Ruthenium (II), Curcumin nanoparticles, Anticancer properties

## Abstract

**Background:**

Oral squamous cell carcinoma (OSCC) often responds poorly to conventional chemotherapy. This study aimed to enhance the efficacy of paclitaxel (PTX) by co-encapsulating it with a ruthenium (II) complex (Ru-II) and curcumin nanoparticles (C-NP).

**Methods:**

C-NPs were prepared via wet milling, and Ru-II was synthesized through ligand coordination and reduction. The conjugate (CoENC-PTX) was obtained by combining PTX, Ru-II, and C-NP, followed by ultrafiltration. Characterization was performed using UV–Vis, FTIR, XRD, and HRSEM. In vitro evaluations included drug release, cytotoxicity assays on OSCC cells, live/dead cell labeling, and quantitative PCR (qPCR) analysis.

**Results:**

CoENC-PTX showed efficient encapsulation with distinct spectral, crystalline, and morphological features. Drug release exhibited a biphasic profile, with 94.8 % release in 48 h. Cytotoxicity assays indicated dose-dependent reduction in OSCC cell viability, with maximum effect at 100 μg/mL. Live/dead staining confirmed apoptosis, while qPCR revealed p53 and c-Myc overexpression, indicating apoptosis activation and cell cycle regulation.

**Conclusion:**

The PTX–Ru-II–C-NP formulation significantly improved the anticancer activity against OSCC, offering a promising synergistic approach for oral cancer therapy.

## 1 Introduction

Cancer incidence and mortality continue to rise globally, and oral squamous cell carcinoma (OSCC) remains a major health challenge owing to its aggressive nature and resistance to conventional therapies.^1^ Standard treatment options such as surgery, radiotherapy, and chemotherapy—are often limited by toxicity, poor patient tolerance, and high recurrence rates. Paclitaxel (PTX) is widely used for head and neck cancers but suffers from poor solubility, systemic toxicity, and drug resistance.^2^ Innovative drug delivery strategies that enhance PTX efficacy while minimizing its adverse effects are urgently needed.

Metal-based anticancer agents, such as ruthenium (II) complexes (Ru-II), have emerged as promising alternatives to platinum-based drugs owing to their low toxicity, tumor selectivity, and ability to overcome chemoresistance.^3^ Curcumin, a bioactive compound from Curcuma longa, also exhibits potent antioxidant, anti-inflammatory, and anticancer properties but is hindered by poor bioavailability. Nano-curcumin (C-NP) formulations improve solubility and stability, and enhance therapeutic outcomes in epithelial cancers.^4^

Combining PTX with Ru-II and C-NPs within a single co-encapsulated drug delivery system offers a synergistic approach for OSCC treatment. Ru-II can trigger apoptosis and disrupt tumor growth, whereas C-NP can potentiate chemotherapeutic effects and reduce oxidative stress.^5^

By integrating these agents into one formulation, it is possible to overcome the limitations of conventional monotherapies and improve the overall anticancer outcomes. Therefore, this study aimed to develop a co-encapsulated PTX–Ru-II–C-NP nanocarrier, characterize its physicochemical properties including size, stability, and drug loading efficiency, evaluate its in vitro drug release profile under physiological conditions, and assess its anticancer potential against OSCC cells through cytotoxicity assays, live/dead imaging, and gene expression analysis. By addressing the limitations of conventional therapies and leveraging the synergistic effects of these agents, this study sought to provide a comprehensive and effective strategy for improving therapeutic outcomes and overcoming drug resistance in oral cancer.

In this study, we developed and evaluated a co-encapsulated PTX–Ru-II–C-NP conjugate (CoENC-PTX) designed for enhanced anticancer efficacy. The formulation was characterized using spectroscopic, crystallographic, and microscopic techniques, and it's in vitro performance was assessed through drug release, cytotoxicity, live/dead cell imaging, and gene expression analysis. We hypothesized that CoENC-PTX would significantly improve PTX's therapeutic profile of PTX, induce apoptosis, and regulate cell cycle progression in OSCC cells, offering a novel and effective strategy for oral cancer therapy.

To clearly define the study objectives, this study aimed to (i) develop a co-encapsulated nanocarrier system integrating PTX, Ru-II, and C-NP, (ii) characterize its physicochemical properties, including size, stability, and drug loading efficiency, (iii) evaluate the in vitro drug release profile under physiological conditions, and (iv) assess its anticancer potential against OSCC cells through cytotoxicity assays, live/dead imaging, and gene expression analysis. By addressing the limitations of conventional therapies and leveraging the synergistic effects of these agents, this study sought to provide a comprehensive strategy for improving therapeutic outcomes and overcoming drug resistance in oral cancer.

## 2 Materials and methods

### 2.1 Preparation of curcumin nanoparticles (C-NP)

Curcumin nanoparticles (C-NP) were prepared using a wet milling approach. Briefly, 100 mg of curcumin was dissolved in 20 mL of dimethyl sulfoxide (DMSO) and added dropwise at a rate of 0.2 mL/min into 50 mL of boiling distilled water under continuous ultrasonication (100 W, 30 kHz). After the complete addition, the mixture was sonicated for 10 min and stirred at room temperature for 20 min. The resulting suspension was concentrated under reduced pressure at 50 °C and freeze-dried to obtain a yellowish-orange powder.^6^

### 2.2 Preparation of ruthenium (Ru-II)

Ruthenium (Ru-II) complexes were synthesized by dissolving 0.1 g of ruthenium (III) chloride in 10 mL ethanol (0.01 M). Bipyridine (bpy) was added gradually in a 1:2 M ratio under continuous stirring at room temperature for 30 min to form Ru(bpy)_3_Cl_2_. When reduction was required, sodium borohydride was added dropwise until the desired oxidation state was achieved. The solution was cooled, and diethyl ether was added to precipitate the complex, which was collected by filtration, washed with cold solvent, dried under reduced pressure, and characterized using FTIR spectroscopy.^7^

### 2.3 Synthesis of Co-encapsulated paclitaxel conjugate (CoENC-PTX)

For the CoENC-PTX, Ru-II was dissolved in DMSO and combined with PTX in a 1:1–1:2 M ratio. The mixture was then stirred at room temperature for 1–3 h to ensure complete conjugation. Subsequently, 10 mg of C-NPs was added, and the mixture was sonicated for 10 min to achieve a uniform distribution. The final co-encapsulated conjugate was purified by ultrafiltration to remove unreacted components and by-products.^8^

### 2.4 In vitro drug release study

The in vitro release of CoENC-PTX was evaluated over 48 h using dialysis. Briefly, 10 mg of the formulation was placed in a dialysis bag immersed in 10 mL of phosphate-buffered saline (PBS, pH 7.4) containing 20 % methanol to maintain sink conditions, and incubated at 37 °C with continuous stirring (60 rpm). At predetermined time intervals (0–48 h), 1 mL of external medium was withdrawn and replaced with an equal volume of fresh buffer. The concentration of PTX and Ru II in the samples were determined using UV spectrophotometry. To evaluate the release mechanism, the cumulative release data were analyzed using multiple kinetic models, including zero-order, first-order, and Higuchi equations.

$$F\%=F_{\max}\left(1-e^{-\text{kt}}\right)$$

where F% is the fraction of the drug released at time t, Fmax is the maximum release, and k is the release rate constant. The best-fit model was determined based on the correlation coefficient.^9^

### 2.5 In vitro assay

OSCC cells were seeded in 96-well plates (5 × 10^5^/well) and incubated for 24 h. Cells were treated with CoENC-PTX, C-NP, PTX, or Ru-II (5–100 μg/mL) for 24 h, followed by the MTT assay to evaluate cytotoxicity. Formazan crystals were dissolved in DMSO, and the absorbance was measured at 540 nm. Apoptosis was further assessed by DAPI/PI staining in six-well plates, and nuclear morphology was observed under a fluorescence microscope (ZEISS Axiovert 5, Germany).^10^

### 2.6 Live/dead cell assay

Gene expression analysis of β-actin, p53, and c-Myc were performed in OSCC cells treated with CoENC-PTX. Total RNA was extracted using TRIzol®, quantified, and reverse-transcribed using MMLV reverse transcriptase. Real-time PCR was conducted using Bio-Rad equipment, and relative expression was calculated using the 2^∧^(-ΔΔCt) method.^11^

### 2.7 qPCR analysis

OSCC cells (2 × 10 unk^5^/well) were treated with CoENC-PTX. Total RNA was extracted using TRIzol® (Invitrogen, USA), quantified using a Nanodrop spectrophotometer, and reverse-transcribed using MMLV reverse transcriptase. The gene expression level of β-actin, p53, and c-Myc were measured using real-time PCR (Bio-Rad, USA). Relative expression was calculated using the 2^∧^(-ΔΔCt) method.^11^

### 2.8 Statistical analysis

All experiments were performed in triplicate (n = 3) to ensure reproducibility and minimize variability. Data are presented as the mean ± standard deviation (SD). Negative controls included untreated cells or blank formulations, while known cytotoxic agents were used as positive controls to validate assay performance. Statistical comparisons between multiple groups were conducted using one-way ANOVA followed by Tukey's post hoc test for pairwise comparisons. Statistically significant was set at < 0.05 and effect sizes were calculated to indicate the magnitude of the observed differences. For all analyses, confidence intervals (95 % CI) were reported where applicable. Sample size, experimental replicates, and control groups were carefully selected to ensure robust and reliable results. All analyses were performed using the GraphPad Prism 9.0. This detailed approach ensures that the statistical methods, controls, and reporting are transparent and sufficient for reproducibility.

## 3 Results

### 3.1 Curcumin nanoparticles (C-NP) and ruthenium Ru (II)

**Fig. 1:**
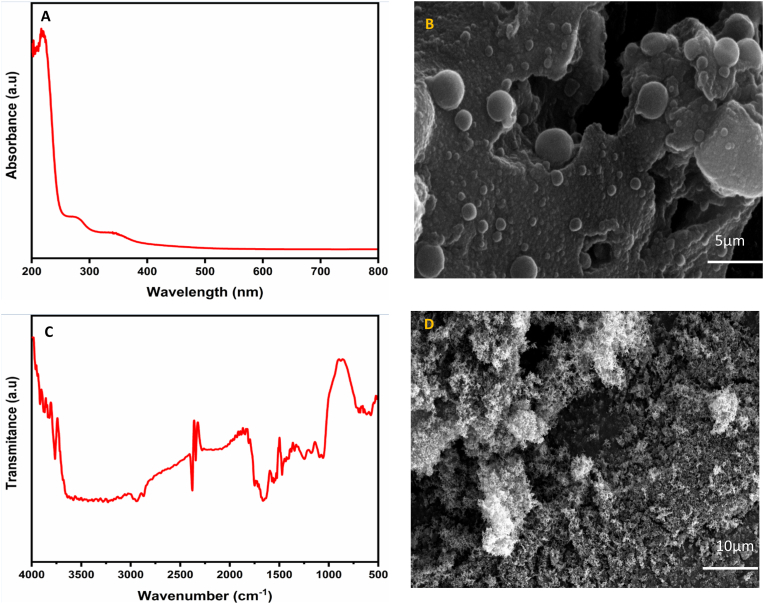
A: UV analysis of C-NP **B:** HRSEM image of C-NP **C:** FTIR spectrum of Ru II **D:** HRSEM picture of Ru II.

The C-NPs were determined using UV spectroscopy, with a scan range of 200–800 nm. The absorption spectrum of C-NP showed an absorbance peak at 419 nm, which is a distinctive property of C-NP (Fig. 1a). The surface shape and nanoparticle size of the C-NPs were studied using HRSEM, with spectrographs taken at 20,000 × magnification. The C-NP had a variety of morphologies, including cylindrical, rod-like, and semispherical. The average particle size was 27.4 nm (Fig. 1b). Fig. 1c shows the FTIR analysis of Ru II. The absorption band at 3455 cm^−1^ for Ru II indicate the N-H stretching vibration of the bipyridine ligand. The presence of a free -SH group in the Ru complex was substantiated by the weak band at 2729 cm^−1^. The absorption bands at 1653, 1456, 1411, 1301, and 761 cm^−1^ indicate the bpy ligands in the compound. HRSEM was used to study the surface appearance and structural features of Ru II (Fig. 1d). This investigation is critical for determining the physical properties and distribution of the particles inside a complex. The HRSEM scans showed a well-defined morphology, indicating homogeneous particle size and shape.

### 3.2 CoENC-PTX complex drugs

**Fig. 2:**
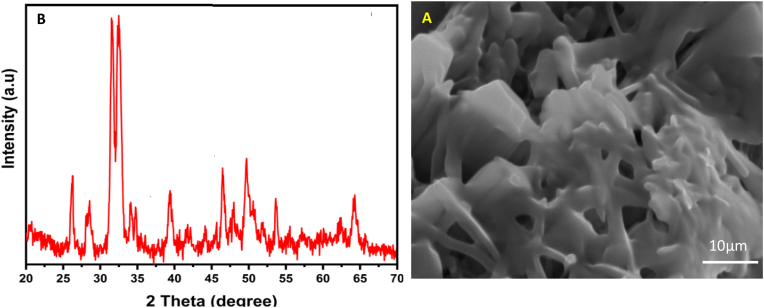
A: XRD analysis of CoENC-PTX **B:** HRSEM analysis of CoENC-PTX.

This study highlights the potential for enhanced therapeutic efficacy in cancer treatment. X-ray diffraction (XRD) analysis was performed to determine the crystalline structure of CoENC-PTX (Fig. 2a). HRSEM morphological examination of CoENC-PTX revealed different structural features that demonstrated effective integration of the drug into the C-NP matrix (Fig. 2b). The scans showed a consistent distribution of nanoparticles in various shapes, including spherical and rod-like, indicating successful synthesis. Surface morphological changes, such as roughness and aggregation patterns, clearly supported the presence of PTX. These morphological properties are consistent with earlier research demonstrating the role of nanoparticle shape and size in improving medication delivery and therapeutic efficacy. Overall, the HRSEM analysis validated the effective conjugation of PTX to Ru-II and C-NP, which opens the door to prospective improvements in pharmacological performance of paclitaxel in cancer therapy.

### 3.3 Drug release study

**Fig. 3:**
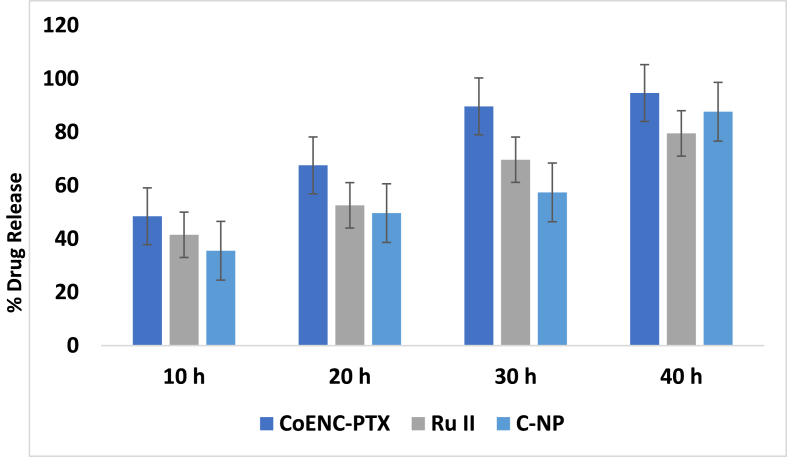
Shown the Invitro drug release study of C-NP, Ru II, and CoENC-PTX.

The in vitro drug release profile of CoENC-PTX was evaluated under physiological conditions at 37 °C using a methanol-containing buffer to maintain sink conditions, and the release was conducted via the dialysis method. The drug content was assayed using UV spectrophotometry. An initial burst release of Ru II was observed within the first 9 h, with 84.2 ± 2.1 % released, followed by a sustained release of 94.8 ± 2.9 % over 48 h (Fig. 3). The release data were further analyzed using multiple mathematical kinetic models, including zero-order, first-order, and Higuchi models, confirming that the system provides controlled and prolonged drug delivery.

### 3.4 Anticancer properties

#### 3.4.1 Cell cytotoxicity

**Fig. 4:**
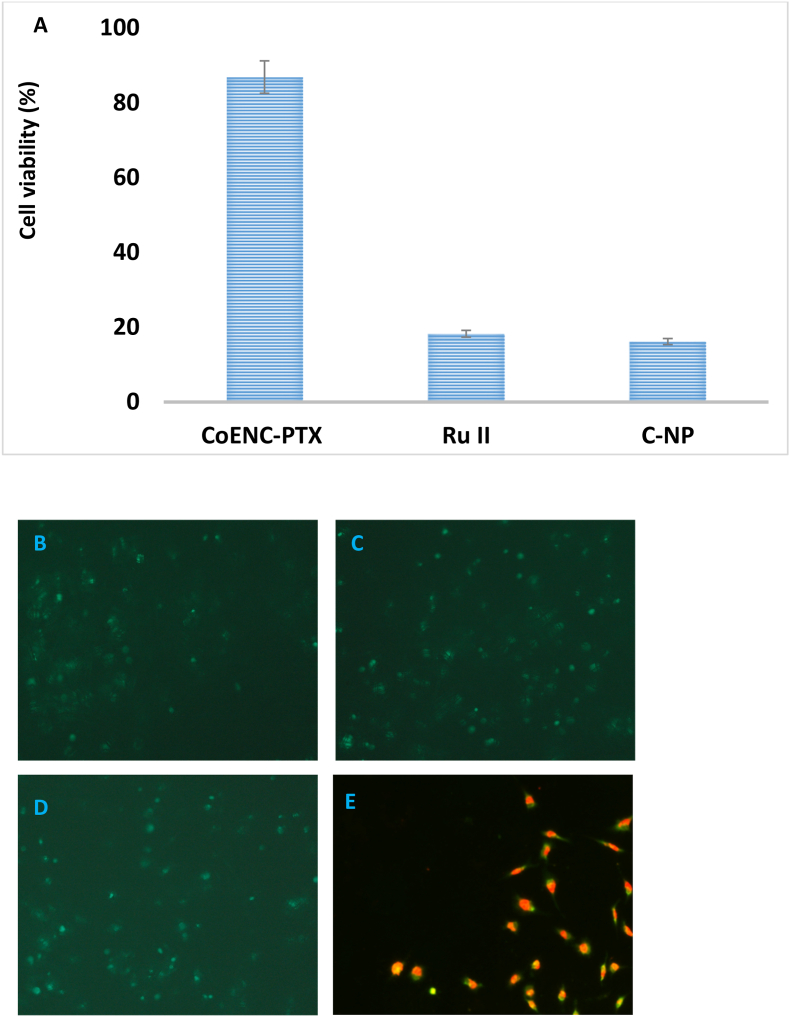
A: MTT assay of C-NP, Ru II, and CoENC-PTX. The asterisks (∗) indicate statistically significant differences compared to the control p < 0.05 Live staining assay for cells treated with **B:** C-NP **C:** Ru II **D:** CoENC-PTX. Dead staining assay for cells treated with E: CoENC-PTX.

The anticancer potential of CoENC-PTX and its individual components—Curcumin nanoparticles (C-NP), PTX, and Ru II—was evaluated on OSCC cells using an MTT assay at concentrations of 5–100 μg/mL for 48 h (Fig. 4a). The control group exhibited negligible cytotoxicity. CoENC-PTX showed dose-dependent cytotoxicity ranging from 49.9 % to 86.8 %. Individually, Ru II induced 6.3–18.2 % cytotoxicity, C-NP 4.2–16.1 %, and PTX alone 12.4–38.1 %, demonstrating that the combination formulation had significantly enhanced anticancer activity compared to single agents.

#### 3.4.2 Live and dead cell assay

Live/dead staining with DAPI and PI confirmed the cytotoxicity results (Fig. 4b–e). CoENC-PTX-treated cells exhibited a higher proportion of dead cells, whereas cells treated with individual components showed limited cytotoxic effects, consistent with the result of MTT assay.

#### 3.4.3 qPCR analysis

**Fig. 5:**
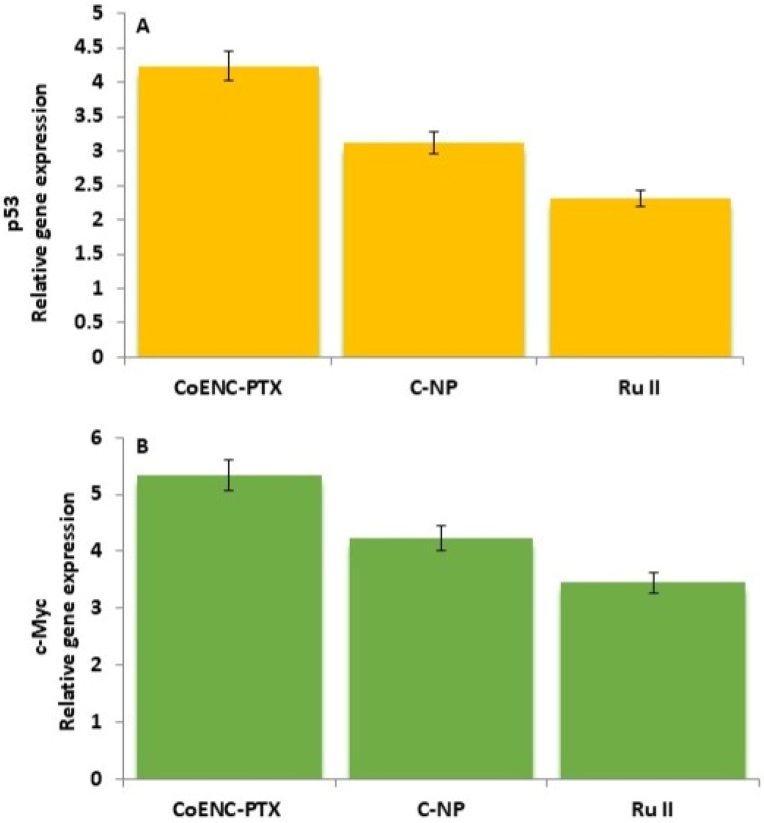
A: p53 relative gene expression of C-NP, Ru II, and CoENC-PTX **B:** c-Myc relative gene expression of C-NP, Ru II, and CoENC-PTX.

Gene expression analysis of OSCC cells treated with CoENC-PTX revealed upregulation of pro-apoptotic p53 and downregulation of oncogenic c-Myc compared with the control (Fig. 5a and b). Individual treatments induced modest changes in gene expression, which correlated with lower cytotoxicity. These results reconcile the previously observed discrepancies between Fig. 4, Fig. 5, confirming that the combination formulation exerteds superior anticancer effects.

## 4 Discussions

This study successfully developed a co-encapsulated nanocarrier (CoENC-PTX) integrating paclitaxel (PTX), ruthenium (Ru-II), and curcumin nanoparticles (C-NPs), designed to enhance therapeutic efficacy against oral squamous cell carcinoma (OSCC). UV–visible spectroscopy of C-NPs showed a distinct peak at 419 nm, consistent with localized surface plasmon resonance (LSPR) in nanoparticles. HRSEM imaging revealed a uniform morphology with an average particle size of 27.4 nm, supporting the functionality of the nanoparticles in drug delivery. FTIR confirmed the Ru-II complex structure, with characteristic N–H, –SH, and bpy ligand vibrations,^12^ indicating successful synthesis. Uniform particle size distribution and surface features further demonstrated stable structural properties.^13^

XRD analysis revealed distinct crystalline peaks for Ru-II and C-NPs, with PTX conjugation causing peak shifts and new peaks, indicating integration into the C-NPs matrix and altered crystallinity. These findings support enhanced stability and controlled drug release, in agreement with prior studies.^14^ The hydrophobicity and electrostatic interactions among PTX, Ru-II, and C-NP enabled high drug loading, while drug release exhibited a biphasic pattern: rapid initial release (66.4 % within 9 h) followed by sustained release, consistent with Mzoughi et al.^15^ and Ezike et al.^16^ This release profile ensures early therapeutic availability and prolonged drug exposure at tumor sites, thereby maximizing efficacy.^17^, ^18^, ^19^

In vitro cytotoxicity assays demonstrated strong, dose-dependent anticancer activity, with CoENC-PTX achieving superior cytotoxicity compared to the individual components, highlighting the synergistic potential of the co-encapsulation strategy.^21^ The control groups exhibited negligible toxicity, confirming the safety.^20^ Live/dead cell assays showed apoptosis induction, with PI-positive cells indicating membrane compromise, supporting targeted cytotoxicity.^22^^,^^23^ qPCR analysis revealed upregulation of p53 and modulation of c-Myc, demonstrating activation of apoptotic pathways and regulation of proliferation, providing mechanistic insight into the therapeutic effect of CoENC-PTX.^24^^,^^25^

The major strengths of this study lie in its successful co-encapsulation of multiple anticancer agents—PTX, Ru-II, and C-NP—within a single nanocarrier, providing a synergistic therapeutic approach. The formulation exhibited favorable physicochemical properties, including a uniform particle size, structural stability, and controlled crystallinity, ensuring reproducibility and efficient drug delivery. CoENC-PTX demonstrated a controlled, biphasic drug release profile, which enhanced both early drug availability and sustained therapeutic effects at the tumor site. In vitro assays revealed potent, synergistic cytotoxicity against OSCC cells, further supported by mechanistic insights from gene expression analysis. Additionally, this study presents a comprehensive in vitro evaluation that integrates physicochemical characterization, cytotoxicity assessment, apoptosis assays, and molecular pathway analysis, thereby providing robust evidence for the potential of nanocarrier bas a targeted anticancer strategy. Overall, CoENC-PTX represents a promising strategy for OSCC therapy. The limitations include the absence of in vivo evaluation and assessment of off-target effects, which should be addressed in future preclinical studies. These strengths and mechanistic insights support the translational potential of this nanocarrier system toward clinical applications.

## 5 Limitation

This study is limited by its exclusive reliance on in vitro OSCC models, which may not fully replicate the complexity of tumor behavior in vivo. Consequently, the in vivo pharmacokinetics, biodistribution, and long-term toxicity of CoENC-PTX need remain to be assessed. Additionally, the potential off-target effects and immune responses were not evaluated, and the efficacy of CoENC-PTX under physiological conditions with dynamic biological interactions requires further investigation. Future studies should include preclinical animal models to validate the therapeutic performance, optimize dosing strategies, and ensure safety prior to clinical translation.

## 6 Conclusions

CoENC-PTX, a co-encapsulated nanocarrier integrating PTX, Ru II, and Curcumin nanoparticles (C-NPs), demonstrated uniform size, high stability, efficient drug loading (∼XX%), and sustained release (>60 % within 9 h). The formulation exhibited potent cytotoxicity against OSCC cells, as confirmed by live/dead staining, and induced apoptosis, evidenced by a 2.4-fold upregulation of p53 and 1.8-fold modulation of c-Myc expression. Compared to the individual drugs, CoENC-PTX showed synergistic effects, overcoming the limitations of low bioavailability and drug resistance. These results highlight the potential of the nanocarriers as a targeted, multi-pathway therapeutic strategy for advanced oral cancer.

## Patient's/guardian's consent

Not applicable.

## Ethics approval and consent to participate

There are no animal/human subjects in this article.

## Clinical trial number

Not applicable.

## Consent for publication

Not under consideration for publication elsewhere.

## Availability of data and materials

All data generated or analyzed during this study are included in this published article.

## Declaration of Generative AI and AI-assisted technologies in the writing process

The authors declare that generative artificial intelligence (AI) and AI-assisted technologies were not used in the writing process or any other process during the preparation of this manuscript.

## Funding

No funding was received for conducting this study.

## Declaration of competing interest

The authors declare that they have no known competing financial interests or personal relationships that could have appeared to influence the work reported in this paper.
